# Improving the Vertical Thermal Conductivity of Carbon Fiber-Reinforced Epoxy Composites by Forming Layer-by-Layer Contact of Inorganic Crystals

**DOI:** 10.3390/ma12193092

**Published:** 2019-09-22

**Authors:** Eunbi Lee, Chi Hyeong Cho, Sae Hoon Hwang, Min-Geun Kim, Jeong Woo Han, Hanmin Lee, Jun Hyup Lee

**Affiliations:** 1 Department of Chemical Engineering, Myongji University, Yongin, Gyeonggi 17058, Korea; 2 Korea Institute of Machinery and Materials, Daejeon 34103, Korea

**Keywords:** carbon fiber-reinforced polymer, inorganic crystals, layer-by-layer coating, mechanical strength, thermal conductivity

## Abstract

A carbon fiber-reinforced polymer (CFRP) is a light and rigid composite applicable in various fields, such as in aviation and automobile industry. However, due to its low thermal conductivity, it does not dissipate heat sufficiently and thus accumulates heat stress. Here, we reported a facile and effective strategy to improve the through-thickness thermal conductivity of CFRP composites by using a layer-by-layer coating of inorganic crystals. They could provide efficient heat transfer pathways through layer-by-layer contact within the resulting composite material. The high thermally conductive CFRP composites were prepared by employing three types of inorganic crystal fillers composed of aluminum, magnesium, and copper on prepreg through the layer-by-layer coating process. The vertical thermal conductivity of pure CFRP was increased by up to 87% on using magnesium filler at a very low content of 0.01 wt %. It was also confirmed that the higher the thermal conductivity enhancement was, the better were the mechanical properties. Thus, we could demonstrate that the layer-by-layer inclusion of inorganic crystals can lead to improved through-thickness thermal conductivity and mechanical properties of composites, which might find applications in varied industrial fields.

## 1. Introduction

Carbon fiber-reinforced polymers (CFRPs) are lightweight continuous carbon fiber composite materials that show high strength [1,2,3]. In addition to their excellent mechanical performance [4,5], they offer the potential to replace heavy metals because of their high corrosion resistance [6]. Therefore, such materials have been applied to a wide range of fields, ranging from the civil/military aviation space industry to the automobile industry, as well as in sports, where lightweight materials are essential [7,8,9,10,11,12]. However, thermoplastic resins with low thermal conductivity limit the commercialization of the CFRP as a polymer matrix for composites [13,14]. Composites with low thermal conductivity do not dissipate heat quickly but accumulate heat, which causes deterioration of the mechanical properties due to peeling between the carbon fiber and the polymer matrix of the composite. The thermal conductivity varies with the direction of the carbon fiber. In a continuous carbon fiber composite material, since the carbon fibers are aligned in the in-plane direction, the vertical thermal conductivity is much lower than that the in-plane direction. Therefore, it is very important to improve the through-thickness thermal conductivity of the composite without deteriorating its mechanical performance.

To improve the thermal conductivity of the composite, it is necessary to improve the internal heat transfer of the polymer matrix, which has lower thermal conductivity and is easier to modify than carbon fiber. One method for improving the thermal conductivity of a polymer matrix is using high-thermal-conductivity fillers, such as graphite and boron nitride [15,16,17,18,19,20], wherein such fillers are mechanically dispersed in a polymer matrix using a mixer or a three-roll mill [21,22,23,24,25,26,27,28,29]. However, in the case of a CFRP, this method is difficult to apply because the carbon fiber is impregnated in a polymer matrix. In the case of a CFRP, the fillers can be included on the carbon fiber fabrics using a bath or direct spray coating process before manufacturing the composite [30,31]. The fillers in the CFRP composite improve the thermal conductivity by providing short heat transfer pathways. However, ensuring an optimal filler content is important for efficient delivery of heat transfer pathways using fillers. If the filler content is too low, it may find it difficult to form heat transfer pathways. On the contrary, if the content is too high, the filler may aggregate to deteriorate the interaction between the filler and the polymer matrix, resulting in deterioration of the thermal conductivity and mechanical properties. It is also important to analyze how the heat transfer interface owing to interactions with the polymer matrix depends on the size and structure of the filler. High interactions of the filler with the polymer matrix can reduce the interfacial resistance between them, thereby efficiently transferring heat.

In this work, we presented a simple and effective strategy for the fabrication of high thermally conductive CFRP composites that simultaneously ensure the excellent vertical thermal conductivity and mechanical strength using layer-by-layer inclusion of inorganic crystals. The three different types of inorganic crystal fillers comprising aluminum, magnesium, and copper were used to study the dependence of the vertical thermal conductivity enhancement on the structure of the inorganic fillers. The CFRP incorporated with inorganic crystal fillers was prepared through a simple layer-by-layer coating process. The inorganic crystal fillers were coated on the surface of the multiple prepregs by layer-by-layer spray coating to lead to layer-by-layer contact of the fillers within the composites. Thus, the high-thermal-conductivity fillers provided a pathway for heat transfer within a low-thermal-conductivity CFRP through layer-by-layer contact so that the composite could dissipate heat efficiently without accumulating it. We analyzed the microscopic surface, size, and structure of inorganic crystal fillers through scanning electron microscope images and measured the vertical thermal conductivity and flexural strength of CFRP coated with inorganic fillers. The thermal conductivity and mechanical properties of CFRP coated with three types of inorganic crystal fillers were compared with those of pure CFRP, and variation in the thermal conductivity behavior and mechanical properties of the CFRP according to the size and structure of the fillers were discussed.

## 2. Materials and Methods

### 2.1. Materials

CFRP prepregs (USN 300BP) consisting of continuous carbon fibers were obtained from SK Chemicals (Seoul, Korea). The prepreg consisted of resin and carbon fiber masses of 33 wt % and 67 wt %, respectively, and was unidirectional type. The resin in the prepreg included diglycidyl ether of bisphenol A epoxy, cyanate ester resin, and a formulated hardener. The carbon fiber was Pyrofil TR50S with a diameter of 6–8 μm and a density of 1.82 g cm^−1^. The fiber areal weight was 300 g m^−3^, and the thickness of one prepreg was 0.288 mm. The inorganic crystal fillers used to improve the vertical thermal conductivity of CFRP were aluminum, magnesium, and copper. Aluminum and magnesium powders of reagent grade were obtained from DUKSAN PHARMACEUTICAL (Ansan, Korea) and DAEJUNG (Siheung, Korea), respectively. In addition, the pure-grade copper powder was obtained from Junsei Chemical (Tokyo, Japan). The dispersion solvent used for spraying inorganic crystal fillers by layer-by-layer coating was extra-pure ethanol and purchased from SAMCHUN (Seoul, Korea).

### 2.2. Layer-by-Layer Coating of Inorganic Crystals

Inorganic crystal fillers were coated on prepregs using an air spray gun. The air spray gun (K-3, BLUEBIRD, Seoul, Korea) used to coat the fillers was connected to an air compressor (KAC-25, KEYANG, Seoul, Korea). Inorganic crystal fillers were dispersed in ethanol before being coated with an air spray gun. The concentration of inorganic crystal fillers dispersed in ethanol was 0.1–4 g/100 mL. The solution was dispersed for 10 min using a digital sonifier (digital sonifier 250, Branson, St. Louis, MO, USA) for homogenization prior to coating. The solution dispersed with a digital sonifier was directly spray-coated on 40 mm × 40 mm prepregs without any chemical treatment. The prepregs coated with the dispersion solution were dried at 30 °C for 30 min to evaporate the ethanol. The weight fraction of inorganic crystal fillers in composites was calculated by weighing the prepregs before and after coating the crystal filler.

### 2.3. Preparation of CFRP Composites

A mold and piston made of steel were used to fabricate CFRP composites of uniform thickness. The sizes of the mold and the piston were 40 mm × 40 mm × 6 mm and 39 mm × 39 mm × 5 mm, respectively, and the CFRP composites produced had dimensions of 40 mm × 40 mm × 1 mm. We laid polyimide (PI) films on the top and bottom of the prepregs to prevent the prepregs from sticking to the mold and piston after they were manufactured at high temperatures and pressures. As a first step, a PI film was laid on a plate made of steel, and the mold was put on a PI film. Then, the four prepregs cut to size were stacked in one direction. The second step was to place the piston on the PI film on top of the stacked prepregs and press the prepregs up to the height of the mold. As a final step, CFRP composites were prepared by pressing prepregs at 200 °C and 5 bar for 2 h using a hot press (M, CARVER, Wabash, IN, USA). The CFRP composites coated with inorganic crystal fillers were prepared in the same way, with only the top prepreg being prepared without any filler.

### 2.4. Characterization

The particle structure of the inorganic crystal fillers was determined by scanning electron microscopy (SEM, SU-70, HITACHI, Tokyo, Japan) at an acceleration voltage of 15 kV. The inorganic crystal fillers were coated with platinum and characterized by SEM. In addition, the inorganic crystal filler coated on the prepreg surface was evaluated for its aggregation behavior by optical microscopy (OM, BX51, Olympus, Tokyo, Japan). Vertical thermal conductivity of the CFRP composites was measured at 25 °C using thermometers (UT321, UNI-T, Dongguan, China), and hot plates (MSH-20D, DAIHAN Scientific, Wonju, Korea) were used as the heat source. Thermocouples (k-type, Dongguan, China) were used to measure the surface temperature change of the CFRP composites. The specimen (40 mm × 40 mm × 1 mm) was placed on a hot plate at a temperature of 150 °C, and the temperature change of top of the specimen was recorded on UT320 software (v1.0, UNI-T, Dongguan, China). Thermal insulation was used to insulate the sides of the specimen to prevent heat loss. The vertical thermal conductivity was calculated using Fourier’s law of heat conduction using the equation Q = −kA dT/dL, where Q is the heat flux of the hot plate, 11.408 W m^−1^, k is the thermal conductivity in the vertical direction, and A is the area of the specimen. Here, dT refers to the temperature difference of the specimen in the vertical direction, obtained using T_1_ = 150 °C and T_2_ (acquired experimentally), and dL refers to the thickness of the specimen, measured with a Vernier caliper. The specimen size for testing the flexural strength was 40 mm × 4 mm × 1 mm. Flexural strength was measured by a 3-point-bending test according to ASTM D790–03 [32] with a universal testing machine (UTM, LD 5K, LLOYD, Bognor Regis, UK).

## 3. Results

**Figure 1:**
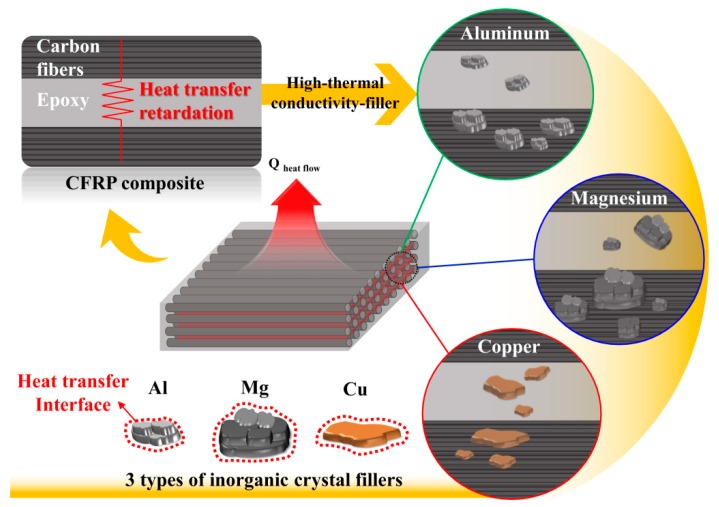
Schematic diagram of the preparation of the high thermally conductive carbon fiber-reinforced polymer (CFRP) composites with inorganic crystal fillers using a layer-by-layer coating.

Figure 1 schematically shows the preparation of high thermally conductive CFRP composites using layer-by-layer inclusion of three types of inorganic crystal fillers. The epoxy polymer matrix of CFRP composites delayed heat transfer owing to its low thermal conductivity. Thus, layer-by-layer contact using high-thermal-conductivity inorganic crystal fillers facilitated heat transfer pathways to the epoxy, which comprised the heat transfer delay zone. The three types of inorganic crystal fillers interacted with the polymer matrix with different heat transfer interfaces depending on the particle size and structure [33,34,35].

### 3.1. Scanning Electron Microscopy (SEM) Analysis

**Figure 2:**
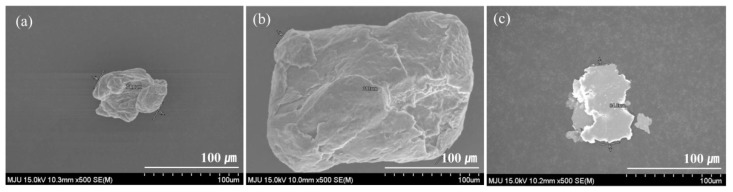
SEM images of three inorganic crystal fillers: (**a**) aluminum; (**b**) magnesium; (**c**) copper.

SEM images of the three types of inorganic crystal fillers are shown in Figure 2. The fillers had different sizes and structures. Aluminum and magnesium showed non-uniform three-dimensional spherical structure. Unlike these two fillers, copper exhibited a flat plate-shaped two-dimensional structure. Aluminum particles exhibited an average diameter of 70 μm, which was the smallest among those of the fillers considered in this work. The average diameter of the largest filler, magnesium particle, was 200 μm. The average diameter of copper particles was 90 μm.

### 3.2. Optical Microscopy (OM) Analysis

**Figure 3:**
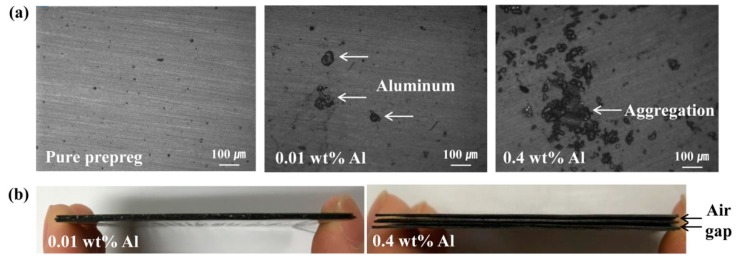
(**a**) Optical microscopy (OM) images of CFRP prepregs: pure prepreg and prepregs coated with aluminum; (**b**) Vertical view of CFRP composites coated with aluminum.

OM images were obtained to determine the aggregation behavior of inorganic crystal filler according to the content. Figure 3 presents optical and photographic images of pure CFRP and CFRPs coated with aluminum filler. Compared to pure CFRP prepreg, the Al-coated prepreg with a high content of 0.4 wt % of aluminum showed large aggregation of the fillers unlike the prepreg coated with a low content of 0.01 wt %, as shown in Figure 3a. In addition, aggregation of the filler that occurred as the content increased deteriorated the interaction with the polymer matrix and caused an interlaminar air gap in the composite, as confirmed in Figure 3b, which shows a vertical view of the CFRP composites. This air gap interfered with the vertical heat flow through the heat transfer pathway formed by the filler and reduced the thermal conductivity increase rate. 

### 3.3. Vertical Thermal Conductivity Analysis

**Figure 4:**
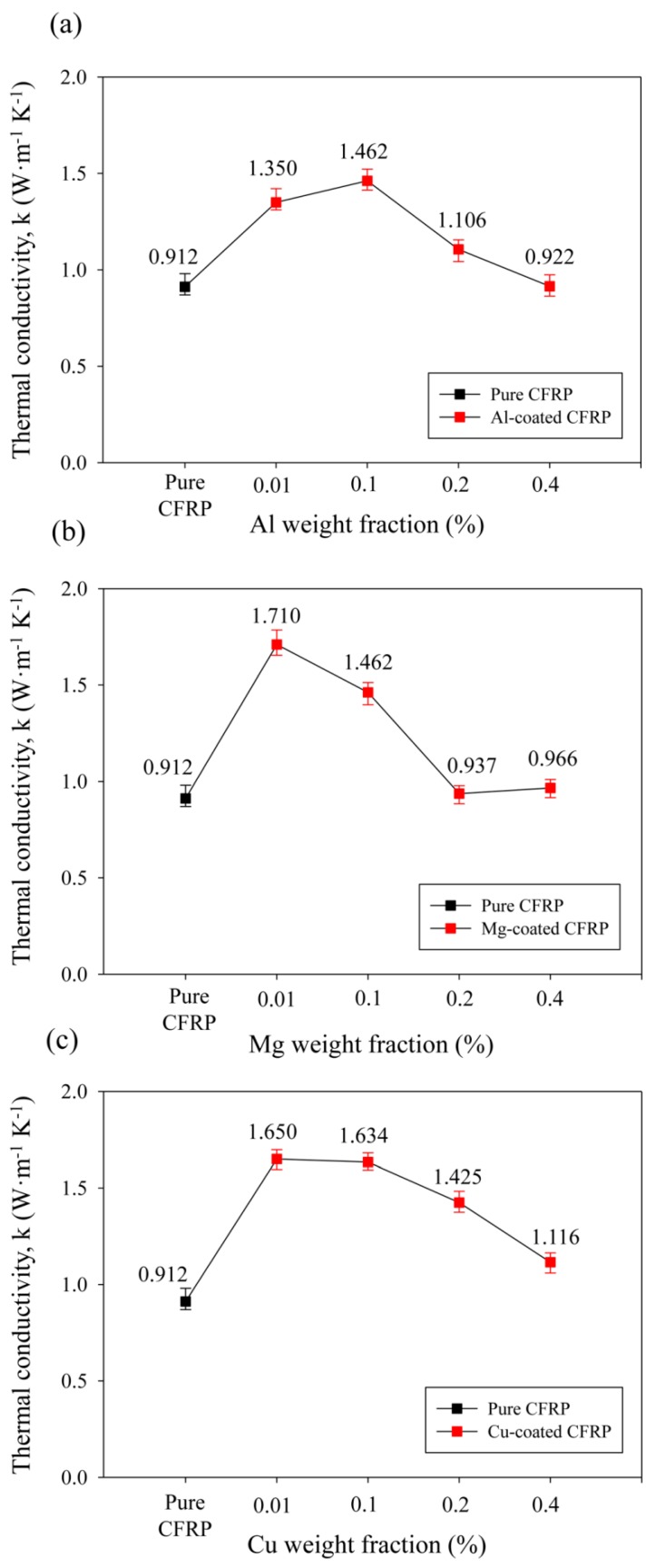
Through-thickness thermal conductivity of CFRP composites coated with three types of inorganic crystal fillers against the filler weight fraction: (**a**) aluminum; (**b**) magnesium; (**c**) copper.

Figure 4 shows the through-thickness thermal conductivity k (W m^−1^ K^−1^) variation according to the contents of the three types of inorganic crystal fillers. Here, pure CFRP refers to a CFRP composite of the same specification as a specimen without any fillers. The vertical thermal conductivity of the pure CFRP was measured to be 0.912 W m^−1^ K^−1^. For CFRP composites coated with inorganic crystal fillers, the thermal conductivity was measured when the content of filler was 0.01, 0.1, 0.2, 0.4 wt % relative to CFRP composites. The inclusion of aluminum filler increased the vertical thermal conductivity with increasing content and showed the highest thermal conductivity of 1.462 W m^−1^ K^−1^ at 0.1 wt %, which demonstrated a 60% improvement over that of pure CFRP. Beyond 0.1 wt %, which led to the highest thermal conductivity, the thermal conductivity enhancement decreased, but the thermal conductivity was still higher than that of pure CFRP. Magnesium filler showed the highest thermal conductivity of 1.710 W m^−1^ K^−1^, which denoted an 87% improvement, at a low content of 0.01 wt %. At the content of 0.1 wt % of magnesium, the thermal conductivity was the same as that of aluminum with the highest thermal conductivity and, beyond that, the thermal conductivity decreased. The copper filler exhibited a vertical thermal conductivity of 1.650 W m^−1^ K^−1^, denoting an improvement of 81% when the content was 0.01 wt %. When its content was 0.1 wt %, the thermal conductivity increased by 1.634 W m^−1^ K^−1^ with the improvement of 79%. At 0.2 wt % or more, the enhancement in the thermal conductivity decreased.

**Figure 5:**
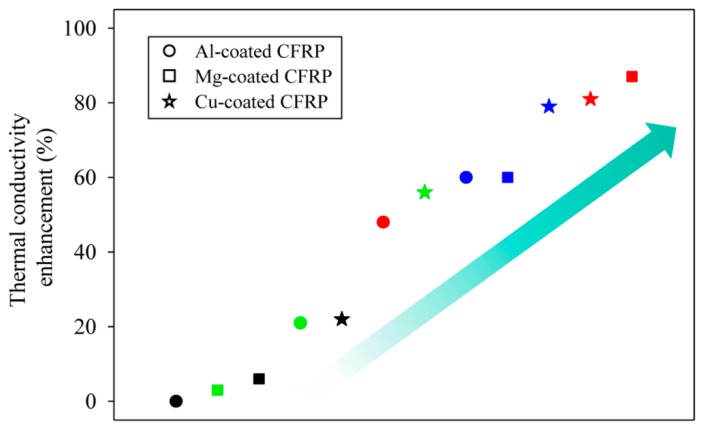
Through-thickness thermal conductivity enhancement of CFRP composites coated with three types of inorganic crystal fillers. The four colors refer to different weight fractions of fillers—red: 0.01 wt %; blue: 0.1 wt %; green: 0.2 wt %, black: 0.4 wt %.

Figure 5 shows the through-thickness thermal conductivity enhancement obtained using three types of inorganic crystal fillers and their contents. Among them, the CFRP coated with 0.01 wt % of magnesium filler showed an 87% improvement in the thermal conductivity at 1.710 W m^−1^ K^−1^ and the highest thermal conductivity enhancement. In addition, CFRPs coated with 0.01 wt % and 0.1 wt % copper exhibited high thermal conductivity enhancements that were lower than that of CFRP coated with 0.01 wt % magnesium.

### 3.4. Mechanical Property Analysis

**Figure 6:**
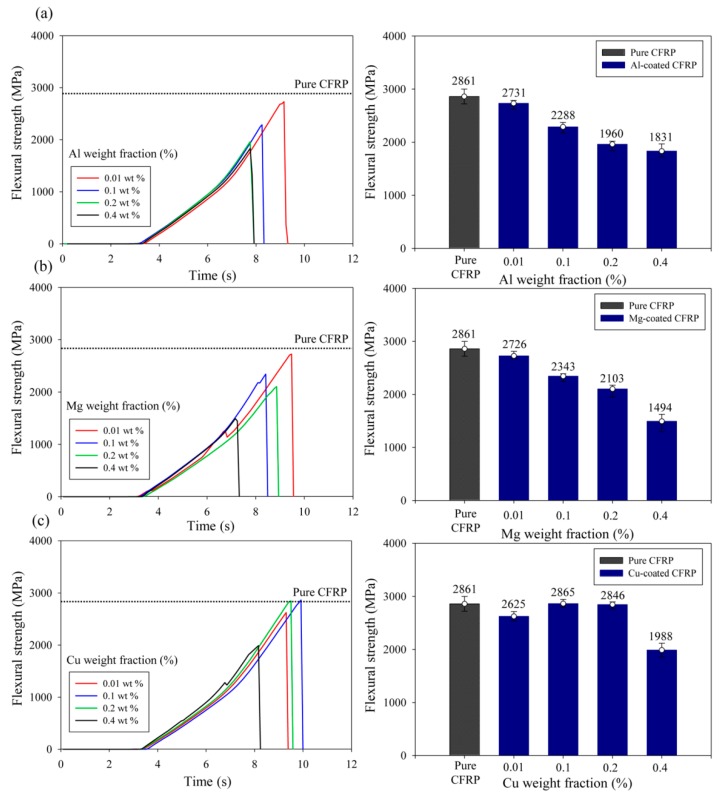
Flexural strength of CFRP composites coated with three types of inorganic crystal fillers: (**a**) aluminum; (**b**) magnesium; (**c**) copper.

Figure 6 shows the flexural strength (MPa) for evaluating the mechanical properties of CFRP composites. The pure CFRP showed a flexural strength of 2861 MPa in the 3-point bending test. The aluminum filler exhibited a flexural strength of 2731 MPa, comparable to that of pure CFRP, at a content of 0.01 wt %, and the flexural strength decreased at contents of 0.1 wt % or higher. The flexural strength of CFRP coated with 0.01 wt % magnesium, which exhibited the highest thermal conductivity enhancement, was 2726 MPa. It was almost equivalent to that of pure CFRP. Moreover, magnesium filler exhibited the same tendency as aluminum with decreasing flexural strength at contents of 0.1 wt % or higher. Copper-coated CFRPs showed no critical degradation of mechanical properties up to a content of 0.2 wt % but showed degraded mechanical properties at 0.4 wt %. 

**Figure 7:**
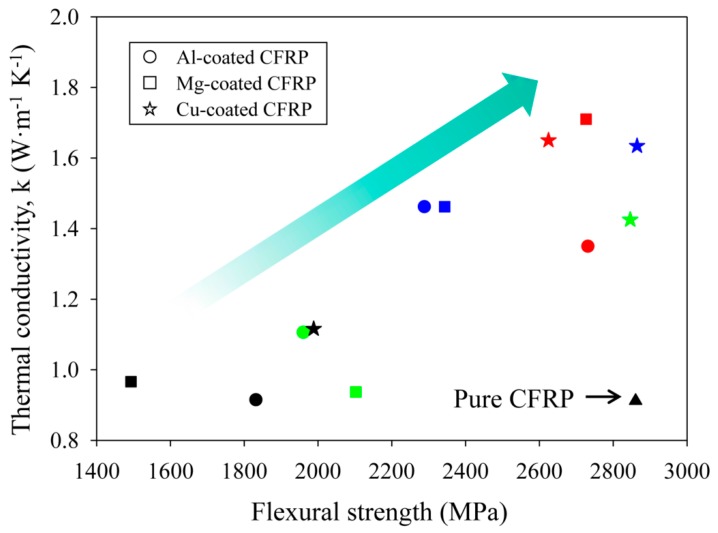
Through-thickness thermal conductivity of CFRP composites coated with three types of inorganic crystal filler against the flexural strength. The four colors refer to different weight fractions of fillers—red: 0.01 wt %; blue: 0.1 wt %; green: 0.2 wt %, black: 0.4 wt %.

Figure 7 shows the vertical thermal conductivity variation according to the flexural strength. In CFRP composites fabricated by a layer-by-layer coating of inorganic crystal fillers, the thermal conductivity and flexural strength tended to increase simultaneously. The CFRP coated with 0.01 wt % magnesium filler showed the highest thermal conductivity as well as high flexural strength.

## 4. Discussion

The vertical thermal conductivity behavior of CFRP composites using layer-by-layer inclusion of inorganic crystals could be rationalized based on the size and structure of the fillers. Aluminum and magnesium fillers have a similar structure with a non-uniform spherical shape, but magnesium particles showed a larger size. When the vertical thermal conductivities of CFRP composites coated with two kinds of fillers of the similar structure but different sizes were compared for each weight fraction, magnesium filler was revealed to increase the thermal conductivities to a greater extent or similarly to aluminum, as shown in Figure 4. While the inclusion of aluminum filler increased the thermal conductivity up to 1.462 W m^−1^ K^−1^ at a content of 0.1 wt %, the incorporation of magnesium filler into CFRP achieved the highest thermal conductivity enhancement at a low content of 0.01 wt %, which represents a 10-fold improvement over aluminum. This could be attributed to the size of the filler particles. Aluminum-coated CFRPs were embedded with small filler particles that could provide more heat transfer pathways than large magnesium filler at the same content when they are homogeneously mixed with the polymer matrix. However, since we used a layer-by-layer spray coating process instead of a filler mixing process, the large size of magnesium was more beneficial in providing a vertical heat transfer path at a low content of filler. Therefore, magnesium and copper fillers increased through-thickness thermal conductivity up to 1.710 W m^−1^ K^−1^ and 1.650 W m^−1^ K^−1^, respectively, at 0.01 wt %, which was 10 times smaller than the 0.1 wt % of aluminum that increased the thermal conductivity to 1.462 W m^−1^ K^−1^.

In addition, the aggregation of inorganic crystal filler at a high content is confirmed in Figure 3: it can lead to degradation of the interaction between the filler and matrix and deteriorate the vertical thermal conductivity and mechanical properties of the composites. When the flexural strengths were compared, as shown in Figure 6, aluminum-coated composites provided better or comparable results in terms of those observed for magnesium-coated composites. The degradation of mechanical property due to agglomeration of the filler was caused by destruction as the force was concentrated on the aggregated portion of the filler. Therefore, when the size of the aggregation was small, the mechanical properties were better [36]. The CFRP coated with 0.01 wt % magnesium filler, which achieved the highest thermal conductivity, showed comparable mechanical properties to pure CFRP due to low aggregation at a low content. Thus, an effective increase in the thermal conductivity at very low contents, such as those of magnesium and copper fillers, is very beneficial.

The copper filler is an inorganic crystal particle with a plate-like structure. It showed a higher thermal conductivity enhancement and flexural strength compared to those arising from other fillers at contents of 0.1 wt % or more. This is because copper filler has a smaller surface area than aluminum or magnesium and is less prone to aggregation and, even if agglomeration occurs, the agglomerated size is smaller than that of magnesium. However, in terms of thermal conductivity properties and cost, improving the vertical thermal conductivity through the low weight fraction of inorganic crystal filler is the most important factor. Therefore, magnesium filler, which showed the highest thermal conductivity enhancement and high mechanical properties at a low content of 0.01 wt %, was considered as the most suitable filler for improving the vertical thermal conductivity of the CFRP composite using a layer-by-layer coating process. Figure 7 presents the correlation between increased vertical thermal conductivity and mechanical properties. This result showed that the flexural strength also increased as the thermal conductivity increased. It was also confirmed through the experimental data that the low content of filler with less occurrence of aggregation was an important factor for maintaining the mechanical strength of pure CFRP. Finally, the CFRP coated with 0.01 wt % magnesium, denoted by red squares, showed the highest vertical thermal conductivity enhancement while maintaining the mechanical strength, compared to the performance of other fillers.

## 5. Conclusions

In this work, we fabricated thermally conductive CFRP composites with excellent vertical thermal conductivity and mechanical strength using a layer-by-layer coating of inorganic crystal fillers. Aluminum, magnesium, and copper—species with different sizes and structures—were used as inorganic crystal fillers. These fillers improved the vertical thermal conductivity of pure CFRP by up to 87%. In particular, magnesium filler led to the greatest increase in through-thickness thermal conductivity at a very low content of 0.01 wt % and also resulted in excellent mechanical property comparable to that of pure CFRP. It could efficiently provide vertical heat transfer pathways because the filler particle diameter was larger than that of aluminum and copper fillers. In addition, since it is spherical, the vertical thermal conductivity could be improved to a greater extent, even at a lower amount than that of the plate-like copper filler. Based on these results, it was confirmed that the shape and size of inorganic crystal filler are important factors to be considered in attempts to improve the vertical thermal conductivity while maintaining the mechanical properties of the CFRP composite through layer-by-layer inclusion of inorganic crystals. This work also provided a basis for expanding the application field of CFRPs by proposing an effective pathway that simultaneously achieves their mechanical properties and vertical thermal conductivity through the use of very small amounts of inorganic crystal fillers.
